# Small molecule screening in zebrafish: an *in vivo *approach to identifying new chemical tools and drug leads

**DOI:** 10.1186/1478-811X-8-11

**Published:** 2010-06-12

**Authors:** Kerrie L Taylor, Nicola J Grant, Nicholas D Temperley, E Elizabeth Patton

**Affiliations:** 1 MRC Human Genetics Unit and the Division of Cancer Research, Institute of Genetics and Molecular Medicine, The University of Edinburgh, Crewe Road South, Edinburgh, EH4 2XR, UK

## Abstract

In the past two decades, zebrafish genetic screens have identified a wealth of mutations that have been essential to the understanding of development and disease biology. More recently, chemical screens in zebrafish have identified small molecules that can modulate specific developmental and behavioural processes. Zebrafish are a unique vertebrate system in which to study chemical genetic systems, identify drug leads, and explore new applications for known drugs. Here, we discuss some of the advantages of using zebrafish in chemical biology, and describe some important and creative examples of small molecule screening, drug discovery and target identification.

## Genetic and chemical screens in zebrafish

Zebrafish (*Danio rerio*) have a unique status in experimental biology. As vertebrates used for forward and reverse genetics, they have provided novel insight into development and disease genetics. More recently, zebrafish research has pushed forward the exploration of chemical biology in a whole animal system. The advantages of using zebrafish as an experimental system for chemical biology mirror those already well established for their use in genetics. Only a few centimetres long as adults, thousands of zebrafish can be housed in a laboratory with relatively low husbandry costs. Breeding pairs can produce over 200 embryos each week that are fertilized outside of the mother and can be easily collected from the breeding tank. Embryonic development from a single cell, and the rapid formation of discrete tissues and organs with physiological similarity to their human counterparts, can be viewed in real time under a light microscope [1]. Organ progenitors can be observed by 36 hpf (hours post-fertilization), hatching occurs at 48-72 hpf and independent feeding by 5 dpf (days post-fertilization). Whole-mount *in situ *hybridization and antibody staining allows for detection of specific RNA or protein expression (or modification) in the developing whole animal, and transgenic technology provides the tools to follow the expression of a specific gene (or series of genes) in the living fish [2]. The zebrafish genome is sequenced, and genetic mutants affect a wide range of biological processes including development, behaviour, metabolism, vision, immunity and cancer.

Genetic screens in zebrafish proceed in two main approaches: forward and reverse genetics [3]. Forward genetics, characterized as 'phenotype to genotype', first involves the identification and characterization of a specific phenotype, followed by the identification of the underlying genetic mutation. The zebrafish system has been especially powerful in the identification of developmental phenotypes, caused by N-ethyl N-nitrosourea (ENU) and insertional mutagenesis, and many of the underlying genetic mutations have been identified [4-8]. Reverse genetics, 'genotype to phenotype', takes advantage of molecular biology techniques. In these cases, a gene of interest is selected and targeted by morpholino oligonucleotide (MO) knockdown, TILLING (Targeting Induced Local Lesions IN Genomes), or zinc-finger nucleases to discover the function of the genetic mutation within the fish [9-14].

**Figure 1:**
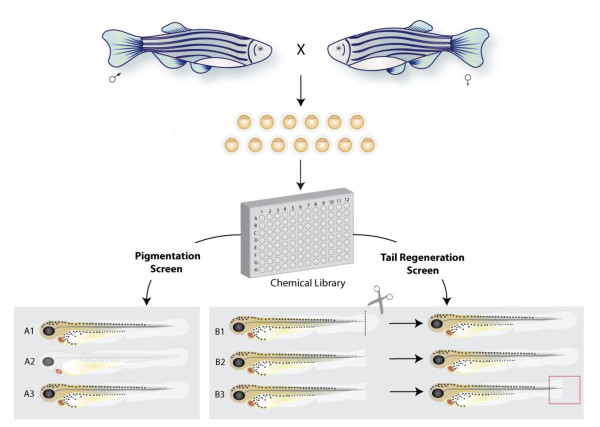
**Phenotype based chemical screening in zebrafish**. Male and female pairs are bred to produce hundreds of single cell embryos that are fertilized *ex vivo*. For high throughput screening, groups of males and females can be bred within in a larger tank (group breeding), producing high numbers of embryos for screening. Breeding is synchronized by the light/dark cycles, and the fish tend to breed within the first two hours of light in the morning. In this example, embryos are arrayed in 96-well plates, each with a different chemical compound, and observed for a specific phenotype. The chemical in well A2 causes a loss of melanocytes, like MoTP [18]. MoTP can specifically kill differentiated melanocytes, and has become a valuable chemical tool to explore melanocyte stem cell biology [42-44]. Other small molecule screens in zebrafish have also identified pigmentation phenotypes [85,86,96]. In another example of a small molecule screen, compounds are screened for inhibition of tail fin regeneration. The tail fin is clipped and grows back within a few days. A zebrafish embryo treated with the compound from well B3, a glucocorticoid, does not correctly regenerate its tail fin [97].

**Figure 2:**
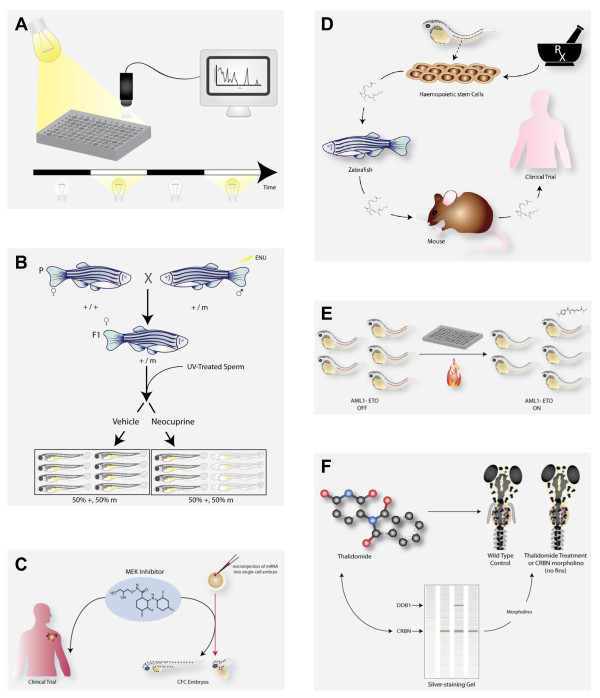
**Creative examples of chemical biology in zebrafish**. The zebrafish system can be used in a wide range of chemical biology experiments and screens. **A**. Zebrafish as young as four dpf have active and sleep-like states. Continuous tracking of movement behaviours of the embryos during rest and wake states, established by light and dark cycles, can be recorded by a camera and computer. High throughput screening for behavioural changes has identified new uses for poorly characterized compounds [80,82]. **B**. Genetic polymorphisms may underlie differences in sensitivity to poor nutrition. In this example, ENU mutagenized zebrafish (parental (P) generation) were screened for genetic mutations that showed sensitivity to sub-optimal copper nutrient conditions. Zebrafish embryos fertilized with UV-inactivated sperm can live as haploid embryos until about 3 dpf [2]. Haploid embryos of the heterozygous mother (F1 generation) were screened for loss of pigmentation, but only in the presence of the small molecule copper chelator, neocuproine [41]. **C**. Intensive efforts by the pharmaceutical industry to develop drugs that target the MAPK pathway to treat cancer patients may also be useful for the management of developmental diseases caused by mutations in the MAPK pathway. In the zebrafish model, expression of BRAF or MEK cardio-facio-cutaneous (CFC) mutant alleles interferes with early development. A one-hour treatment within a specific developmental time window with a MEK inhibitor is sufficient to allow normal development for a CFC zebrafish embryo [75]. **D**. The cardio-vasculature system is conserved in fish, mice and humans. A small molecule screen for changes in hematopoietic stem cells (HSC) development identified the prostaglandin pathway as critical for HSC establishment [59]. A long-acting compound, called dmPGE2, can stimulate HSC development in the embryo and adult zebrafish, as well as in the mouse. dmPGE2 can be safely administered to people, and a clinical trial is underway to see if dmPGE2 treatment of umbilical cord blood prior to transplant can benefit transplant patients (L.I. Zon, *personal communication*). **E**. The acute myelogenous leukemia oncogenic fusion *AML1-ETO (AE) *promotes a change from an erythrocytic fate to a granulocytic cell fate. Erythrocytes express the *gata1 *gene in the posterior blood island of the developing zebrafish (red dotted line). Heat-shock inducible expression of *AE *causes a cell fate change that can be visualized by loss of *gata1 *expression. A chemical screen identified that COX-2 inhibitors can suppress the AE cell fate change, and the embryos maintain *gata1 *expression in the presence of AE [72]. **F**. Zebrafish can play a valuable role in testing for drug toxicity and teratogenisity, as well as for testing direct chemical targets *in vivo*. Thalidomide is a valuable drug for multiple myeloma and leprosy, but caused severe developmental birth defects when taken by pregnant mothers in the late 1950s and early 1960s. Zebrafish are also sensitive to thalidomide, and treatment in early development prevents the proper development of embryonic fins [88]. Thalidomide binds CRBN, and knockdown of *crbn *in the developing zebrafish also causes a loss of fin phenotype. This suggests that the binding of thalidomide to CRBN *in vivo *may underlie its teratogenisty.

Chemical genetics complements traditional genetic approaches [3]. First, many small molecule libraries are made up of compounds with known biological function, allowing rapid elucidation of biological pathways within the organism. Second, chemical treatment can occur at any point during development or in the adult organism, allowing for the study of latent effects of genes during development. Third, chemical dosage can be controlled, which can be advantageous when studying essential functions, or tissue specificity. Small molecule screening identifies relevant targets within the physiological context of the organism, biasing the screen for compounds that are more likely to be cell permeable, less toxic, effective and with an aceptable pharmacokinetic and pharmacodynamic profile [15]. Like genetics, chemical genetics can achieve both forward and reverse approaches [3,15]. In forward chemical genetics, 'phenotype based small molecule discovery', a library of inhibitors can be screened for a specific phenotype in the animal, and from this the target(s) can be identified (Figure 1). Here, we describe some of the phenotype based chemical screens in zebrafish that are pushing forward our knowledge of stem cells, behavior, development and disease treatment (Figure 2A, B, D, E). Conversely, reverse chemical screening entails testing chemical inhibitors with known molecular targets for specific phenotypes in the zebrafish. Examples of this application of the zebrafish system include identifying and optimizing new bioactive compounds, applying a cancer drug to developmental disease, and revealing the teratogenic mechanisms that hinder valuable clinical drugs (Figure 2C, F).

## Considerations of chemical libraries and methodology

In genetic screens, the type of mutagen (*e.g*. ENU, x-ray, insertional mutagen) will determine the range of possible genetic mutations and phenotypes, as well as the ease of identifying the mutation [2,10]. For example, ENU mutagenesis can generate hypomorphic, temperature sensitive, and gain- and loss-of-function mutations that require genetic mapping to identify the genetic lesion. Insertional mutagenesis is more likely to cause gene disruptions, and allows for the rapid identification of the insertion in the genome. Likewise, in chemical biology, the choice of chemical library directs the type of molecular processes that are disrupted, as well the approach of target pathway identification [16]. Traditionally, the discovery of new drugs has been achieved through the isolation of natural products from plants and microbes [17]. Screening a library of natural products will be rich in chemical diversity and may identify novel active compounds, but identifying the active compound(s) and subsequent targets may require extended fractionation procedures and biochemical techniques. Conversely, screening a library designed to target specific classes of enzymes, such as kinases, allows for rapid identification and direct testing of the chemical target *in vivo*, but represents a much smaller range of chemical diversity. Similarly, screening a library of compounds of pharmacologically active molecules of known bioactivity, while again covering a reduced chemical space, may allow for a rapid transition to pre-clinical mammalian models.

As in genetic screens, an important feature of chemical screening is consideration for the screening assay, that is, the phenotypic feature that is the basis for the screen. The zebrafish system allows for diverse screening assays including development and function of internal tissues and organs in living animals that can be illuminated by florescent reporter genes or molecules, or disrupted by genetic mutations. Whole-mount RNA *in situ *hybridization and antibody staining provide details of cellular changes in fixed embryos. No other vertebrate is as well positioned for high throughput phenotyping as the zebrafish embryo [3]. For high-throughput screening, computerized detectors can rapidly screen thousands of treated embryos in a small treatment volume (*e.g*. in a 384 well or 96 well plate), while lower throughput screening can often involve a single investigator screening multiple characteristics in larger treatment volumes (*e.g*. in a 24 well plate).

In the first chemical screen in zebrafish, Peterson and colleagues (2000) screened 1,100 compounds selected from the DIVERSet E Library (Chembridge) in 96-well plates for small molecules that caused developmental phenotypes during the first three days of development [18]. This was an important proof-of-concept study showing that small molecule screening in zebrafish could identify chemicals that, like genetic mutations, disrupt specific developmental processes. In addition, the identified chemicals could be used at different doses and at different developmental intervals to identify the timing of the chemical action during development. Recently, a thorough review of the zebrafish chemical screens performed and libraries used has been published [16]. Here, we describe selected screens and chemical-genetic analyses performed in zebrafish to highlight the range of experimental screen designs and outcomes (Figures 1 and 2).

## Chemical screening to rescue disease phenotypes: aortic coarctation

Many genetic mutations identified in zebrafish are analogous to disease genotypes in humans, or cause phenotypes that share clinical features with human diseases [19,20]. The zebrafish *gridlock *mutant suffers from a malformed aorta that prevents circulation to the trunk and the tail, similar to coarctation of the aorta in humans. The *gridlock *phenotype results from a mutation in the *hey2 *gene, a transcriptional repressor that determines angioblast differentiation [21,22]. To screen for chemical suppressors of aortic coarctation, Peterson and colleagues treated g*ridlock *mutant embryos with small molecules from the DIVERSet E library (Chembridge) from early gastrulating embryos until 48 hpf [23]. From over 5000 chemicals screened, two structurally related compounds suppressed the *gridlock *phenotype. Altering the treatment time during development revealed a critical chemical treatment window for correcting the aorta phenotype to be between 12 and 24 hpf. Importantly, the correction mechanism was identified to be via upregulation of vascular endothelial growth factor (VEGF), and the subsequent promotion of blood vessel development. Small molecule inhibitors of the vascular endothelial growth factor receptor (VEGFR) can inhibit blood vessel formation during zebrafish development, and tail fin regenerative angiogenesis in the adult [24,25]. The chemically induced upregulation of VEGF was also effective at stimulating endothelial cell tubule formation [23]. Thus, phenotypic-based screening can identify suppressors of a genetic disease phenotype that have relevant biological activity in mammalian cells. Importantly, in this example, the target compounds did not affect the disease gene or protein directly. Rather, a pathway critical to the rescue of the phenotype was successfully identified, and can now be considered for new therapeutic approaches.

## Chemical screening to reveal new insight into retinal blood vasculature

A highly metabolic tissue, the retina is fed oxygen and nutrients by an intricate vascular network. In humans, disruption of the retinal vasculature network, through damage to the existing network or inappropriate neovascularization, can lead to loss of vision and severe forms of blindness. Understanding the basic biology of retinal vasculature is critical to understand the aetiology and pathology of retinal disease. In the zebrafish embryo, this has been done through the identification and detailed characterization of genetic mutants which have a disruption of the retinal vasculature network [26]. These studies are facilitated by the generation of transgenic zebrafish lines, expressing green florescent protein (GFP) under the promoter of the endothelial vessel specific *fli1 *gene [26,27]. As a complementary approach to genetics, and to learn about retinal vascularization, the *fli1-GFP *transgenic line was used in a screen for small molecules that could modulate the retinal vascular network during days 3-6 of development [28]. Approximately 2000 small molecules from the bioactive Spectrum library were added to the developing embryos at the pectoral fin stage (approximately 60 hpf), and embryos were then embedded in methyl cellulose and visualized under an inverted microscope. Of the 2000 compounds screened, five displayed a reproducible effect on retinal vessel morphology: two affected vessel diameter without affecting vessel number, one affected vessel diameter and number, and two caused severe collapse and loss of over 80% of the vessels. Extending these studies, Kennedy and colleagues screened a panel of known small molecule regulators of angiogenesis, and found that LY294002, an inhibitor of PI3 kinase signalling, can prevent the ocular angiogenesis in wild type fish and partially correct the extraneous angiogenesis in the *out of bounds *zebrafish mutant [29]. Treatment with LY294002 within specific developmental time-windows, showed that the chemical could affect the development of new vessels, without affecting existing intraocular vessels or retinal function. Importantly, direct and localized delivery of the drug was effective at inhibiting intraocular angiogenesis without additional system effects or altering visual function. Additional studies have shown that chemical modulation in the adult zebrafish can reduce hypoxia induced neovascularization [30]. Studies such as these provide a groundwork for identifying chemicals and chemical methodologies that can specifically target aberrant retinal vascularization, which affects the sight of millions of people worldwide.

## Parallel *in vitro *and *in vivo *chemical screening: identifying new and known cell cycle regulators

Many small molecules that affect the cell cycle have been identified through screening for compounds that alter the cell cycles of cancer cells in culture. Such high-throughput screens allow for large numbers of compounds to be screened on multiple cell lines in a rapid and efficient manner [31]. In the 1960s, cell based screening of natural products by the National Cancer Institute identified taxol as a potent mitotic inhibitor [32]. Derived from the Pacific yew tree, taxol is now considered one of the most important chemotherapeutics to treat breast, lung, and ovarian cancer [33]. Many mammalian cell cycle genes are conserved in zebrafish [34], and the majority of cell cycle inhibitors used in mammalian cells share sufficient conservation with the drug targets to be effective in zebrafish embryos or cell lines [35]. The zebrafish *crash & burn (crb) *mutant for the *bmyb *gene, is a MYB family transcription factor involved in cell cycle progression and cancer. The *crb *mutant was identified in a genetic screen for changes to the cell cycle, as revealed by an increased number of mitotic cells marked by a phospho-histone H3 antibody [36]. *crb *mutants also reveal abnormal spindle and centrosome formation, and polyploidy. Notably, adult zebrafish heterozygous for *crb *have an increased incidence of carcinogen-induced cancer [36]. Using the DIVERSet E library (Chembridge) of 16320 compounds, Zon and colleagues screened for small molecules that could specifically rescue the *crb *phospho-histone H3 mitotic defect [37]. One compound, called persynthamide, was identified that suppressed the mutant phenotype. In wild type embryos persynthamide transiently delays S-phase *via *an ATR dependent checkpoint. Comparison with other known S-phase inhibitors, such as hydroxyurea and amphidicolin, showed that it was the chemically induced S-phase delay that was sufficient to rescue the *crb *defect. In human cells, loss of B-MYB leads to reduced levels of *cyclin B1 *[38], and expression of *cyclin B1 *is sufficient to rescue *crb *mutant zebrafish embryos [36]. The persynthamide-induced S-phase checkpoint also causes an increase in *cyclin B1 *expression and thereby rescues the *crb *mutant. This is another example of phenotypic based chemical screening that rescues a genetic phenotype by targeting the altered pathway, and not the mutated protein product directly.

A common question in chemical screening is whether the identified compounds are specific to zebrafish, and how the results of zebrafish chemical screening compare with mammalian cell line based chemical screens. In the small molecule screen for suppressors of *crb*, additional compounds were identified that disrupted mitosis in wild type zebrafish [35]. While the chemical library had been previously screened extensively for cell cycle inhibitors in mammalian cells, 14 novel compounds that affected the cell cycle were identified *via *screening on zebrafish embryos. Six of the 14 hits were effective in mammalian cell lines, confirming the conservation of the affected target pathways across vertebrate species. Of the remaining seven hits, three were serum-inactivated which accounts for their lack of activity in cell culture systems. Four hits were only active in the zebrafish embryo, but not cell lines, possibly due to activation by yolk sac proteins. One hit was active in zebrafish embryos and zebrafish cell culture, but not in mammalian cell culture, suggesting the target of the compound was specific to the zebrafish and not conserved in mammalian cells. Thus, *in vivo *and *in vitro *chemical screens are complementary approaches, that when used together constitute a powerful approach to identifying a more complete set of chemically bioactive tools.

## Using chemicals to identify gene-nutrient interactions

The ability to limit the timing and regulate the dose of small molecules has allowed for unique insight into the tissue-specific requirements for copper in the developing embryo. Copper is an essential nutrient, and sub-optimal copper nutrient conditions in humans can lead to severe clinical symptoms including disorders of the nervous system, hair, and skin [39]. While copper is essential for all cells, some cells and tissues have a specialized copper requirement. Gitlin and colleagues treated developing zebrafish embryos with the small molecule copper chelator, neocuproine, and found that loss of cuprodependent enzyme activity specifically affected some tissues, including melanocytes, the notochord, and the developing hindbrain [40]. Supported by a genetic mutant in the copper transporter *atp7a *(that was cloned in part by virtue of phenotypic features indentical to the effect of the copper chelator neocuproine), Gitlin and colleagues revealed the time, dose and tissue specific requirements for copper dependent enzymes during embryogenesis [40].

Humans with mutations in *ATP7A *develop Menke's disease, a rare X-linked disorder of copper metabolism, characterized by neurodegeneration, hypotonia, and hypopigmentation. Less well understood are other genetic conditions that lead to sensitivity to sub-optimal copper nutrient conditions in otherwise healthy individuals. To address this problem, the Gitlin group used low doses of neocuproine and screened for genetic mutations that revealed a copper deficiency phenotype under mild copper deficiency conditions (Figure 2B). Two genetic mutants were found: a hypomorphic allele of *atp7a *and mutation of the vacuolar atpase *atp6*. Vacuolar Atp6 is required for proton transport in the secretory pathway, an important process in intracellular copper transport [41]. Thus, genetic polymorphisms that otherwise have normal developmental features can be combined with chemicals to reveal selected sensitivities to sub-optimal nutrient conditions.

## Chemical-genetic and chemical-chemical screens to probe melanocyte regeneration pathways

MoTP (4-(4-morpholinobutylthio)phenol), identified in the original small molecule screen by Peterson and colleagues (2000), caused a dramatic loss of melanocytes by cell death [18]. Tyrosinase is a copper dependent rate limiting enzyme for melanin pigmentation in melanocytes, and MoTP exerts melanocyte-specific cell death *via *its conversion to a cytotoxic form in cells that express high levels of tyrosinase [42]. With a melanocytotoxic compound in hand, the Johnson group has been able to use MoTP to gain significant understanding of melanocyte regeneration in zebrafish. Many tissues and cells, including melanocytes, can regenerate in zebrafish, providing important insight into tissue-specific stem cells and progenitor development. Using MoTP to ablate embryonic melanocytes, ENU mutagenized zebrafish were screened for mutants that fail to regenerate melanocytes after treatment [43]. Two mutants were identified, *eartha *and *julie*, that only regenerated about 10% of the normal complement of melanocytes, affecting late stages of melanocyte differentiation in the regeneration process, and proliferation of melanoblasts during early melanocyte regeneration respectively. Thus, by using a chemical to precisely control a specific cell type and process, genetic mutations necessary for distinct aspects of melanocyte regeneration can be identified.

In a further small molecule screen for compounds that specifically blocked melanocyte regeneration following MoTP-induced melanocyte ablation, Johnson and colleagues identified ICI-118,551, a chemical that specifically blocks melanocyte regeneration, while having no effect on ontogenetic melanocyte development and other developmental processes [44,45]. One target of ICI-118,551 is the β2-adrenergic receptor, but it is not clear if this is the mechanism by which it blocks melanocyte regeneration. Nonetheless, it is still a useful tool to probe the origin of melanocytes. For example, transgenic zebrafish expressing kit ligand have an increased number of ontogenetic melanocytes, that are suppressed by ICI-118,551 treatment, indicating that kit ligand exerts its effects on the stem cell lineage of the developing zebrafish [44,45].

The genetic mutant, *picasso *develops normal larval melanocytes (ontogenetic melanocytes), but shows deficits in forming new melanocytes at the onset of metamorphosis. Positional cloning identified the mutation causing the *picasso *phenotype to be in the *erbb3b *gene, an epidermal growth factor receptor (EGFR)-like tyrosine kinase [45]. Sequential death and regeneration cycles by successive MoTP treatments in *picasso *embryos showed that they had a defect in their capacity to regenerate melanocytes [44]. Treatment of wildtype embryos with a commercially available Erbb inhibitor mimics the *picasso *phenotype of abnormal adult melanocyte development and melanocyte regeneration after MoTP ablation.

Thus, through a combination of chemical screens and genetics, Johnson and colleagues have identified two existing populations of melanocytes that contribute to zebrafish pigmentation: ontogenetic erbb3b-independent melanocytes that develop within the first 72 hpf, and an erbb3b-dependent melanocyte progenitor population that is laid down before the first 48 hours of development and provides the adult melanocytes, as well as the adult and larval regenerating melanocytes following ablation. In humans, melanocyte stem cells maintain hair color, and cancer stem cells may support melanoma development and chemoresistance. A series of chemical tools that control distinct aspects of melanocyte biology in zebrafish will be valuable tools to test in zebrafish and mammalian models of melanoma [46-48].

## Gene-drug interactions that modulate mechanosensory hair cell death

Hearing loss caused by the death of inner ear sensory hair is a common medical problem. Over a third of ageing people have significant hearing loss, and younger people can also suffer irreversible hearing loss after antibiotic or chemotherapeutic treatments. In zebrafish, the lateral line helps the fish to detect changes in water pressure and water movement, and shares structural and functional similarities with mammalian inner ear hair cells [49]. The developing lateral line system in zebrafish consists of mechanosensory hair cells supported by rosette-like structures of neuromasts [50,51]. Like mammalian ear hair cells, treatment with aminoglycoside antibiotics, such as neomycin and gentamicin, can cause mechanosensory hair cell death [52,53]. Using vital dyes that allowed for visualization of the living neuromasts, Raible and colleagues screened over 10,000 small molecules from the DIVERSet E Library (Chembridge) for compounds that protected the mechanosensory hair cells from neomycin toxicity [52]. Two compounds, PROTO-1 and PROTO-2 (benzothiophene carboxamides) were identified, that showed significant protection from the ototoxic effects of neomycin. Importantly, when combined with neomycin in microbiological testing, PROTO-1 and PROTO-2 did not interfere with neomycin's antibacterial activity. Aminoglycoside drug uptake depends on ion channel activity which can be significantly blocked by altering extracellular calcium concentrations [54]. Thus, a class of small molecules has been identified that appear to specifically protect the mechanosensory hair cells from death in the presence of aminoglycosides. These compounds have relevance for the mammalian inner ear because they show some protection against neomycin induced hair cell loss in an *in vitro *mouse utricle preparation [52].

As with neocuproine and MoTP, one of the exciting avenues for chemical biology in zebrafish is the potential to screen for genetic mutations that reveal a specific phenotype in the presence of the drug (gene-environment interactions). Raible and colleagues extended their chemical screening to identify genetic mutations that protect against the effects of neomycin induced hair cell death (Owens et al. 2008). Five recessive loci that were either specific to neomycin resistance or have additional phenotypes were identified. One mutant, called *sentinel*, had a mutation in a novel and conserved gene that had not previously been functionally studied in an animal system. Notably, neither PROTO-1 nor *sentinel *could protect against another ototoxic compound, cisplatin, suggesting that PROTO-1 and *sentinel *act specifically to prevent aminoglycoside toxicity. Hearing loss is variable in the human population, and polymorphisms are associated with familial, environmental and drug induced adult-onset hearing loss [55-57]. Through screening, zebrafish have a unique role in the identification of chemical and genetic modifiers of drug-induced hearing loss. Already, additional screening of an FDA-approved small molecule library for those that protect zebafish lateral lines hair cells has identified tacrine as a drug that protects the mouse utricle from neomycin induced hair cell death [52,58].

## Screening for new applications of clinical compounds: hematopoietic stem cells

There are now numerous examples of small molecules that show effects in zebrafish and have relevant bioactivity in mammalian cells. But will the knowledge gained in the zebrafish system be directly translatable to the clinical setting? Zon, North and colleagues have used chemical screening in the developing zebrafish to identify small molecules that modify hematopoietic stem cell (HSC) number *in vivo *[59]. Definitive HSCs develop in the aorta-gonad-mesonephros (AGM) region, and then colonize the hematopoietic organs [60]. In zebrafish and mammals, *runx1 *and *cmyb *genes are expressed in the AGM and are required for HSC formation. Small molecules were screened by RNA *in situ *hybridization for those that altered *runx1 *and *cmyb *expression in the AGM (Figure 2D). Of 2480 compounds screened, 82 compounds were identified that affected HSC number, and of these ten were small molecules with known effects in the prostaglandin pathway [59]. The prostaglandins are evolutionarily conserved lipid signaling molecules that are derived from arachidonic acid, that are first processed by the cyclooxygenases (COXs) and then further by prostaglandin synthases (PGEs) to generate the effector prostaglandins, such as PGE2 [61]. PGE2 signals through G-protein coupled receptors and plays an important physiological role in smooth muscle contraction and relaxation, pain, inflammation and blood clotting. COX inhibitors are widely used as non-steroidal anti-inflammatory drugs, the most common being aspirin and ibuprofen. Confirming the targets of the small molecules identified in the screen, morpholino oligonucleotide knockdown of *cox1 *and *cox2 *reduced HSC formation, an effect that could be rescued by the addition of a long acting derivative of PGE2 (dmPGE2). Likewise, knockdown of the PGE2 receptors diminished HSC *runx1 *and *cmyb *expression.

With HSC homeostasis under effective chemical control in embryos, Zon and colleagues then wanted to establish if prostaglandin signaling was effective in adult fish and conserved in mammals (Figure 2D). In adult zebrafish, the site of hematopoiesis is in the kidney, and dmPGE2 was shown to be effective at stimulating HSC dependent kidney marrow recovery in irradiated wild type fish [59]. Next, in mice, whole bone marrow (WBM) was stimulated *ex vivo *by dmPGE2 before being transplanted into irradiated recipients, and was found to increase hematopoietic progenitor formation. Administration of dmPGE2 also enhanced bone marrow recovery after 5-fluorouracil induced bone marrow injury, and conversely, administration of COX inhibitors diminished WBM and blood recovery. In this way, these studies have found an important and druggable regulator of HSC homeostasis that is conserved in vertebrates. Patients that have depleted HSCs may benefit from dmPGE2 stimulated cord blood transplants as a therapy to expand HSCs and enhance engraftment [61]. Based on these studies, dmPGE *ex vivo *treatment is currently in clinical trial for patients receiving cord blood transplants [61]; L.I. Zon, *personal communication*).

The versatility of the zebrafish assay and the evolutionary conservation of zebrafish biology has allowed further work using prostaglandin chemical modulators to tease apart the developmental pathways that reguate HSCs. Wnt signalling is critical for development, regeneration and stem cells [62-64]. Using a transgenic β-catenin-responsive reporter line, dmPGE2 treatment increased Wnt actvity in the AGM that co-localized with HSCs [62]. Further detailed studies using a combination of chemical modulators, the β-catenin-responsive reporter line, and fish and mouse adenomatosis polyposis coli (APC) mutants strongly supported a conserved PGE2-Wnt signalling axis in development of HSCs. This axis also appears to play a critical role in liver regeneration in both adult fish and mice, and may be a general coordinated proliferative / anti-apoptotic response to wound healing [62]. *In vitro *experiments also suggest a close relationship between Wnt signalling and PGE2 in cellular proliferation and onocogenesis in colon cancer cell lines [65,66]. These results bear clinical significance because COX inhibitors, such as aspirin, reduce PGE2 levels, and also are known to reduce the number and size of colorectal adenomas in colon cancer patients [67].

Heartbeat and circulation is present in early developing zebrafish embryos, despite sufficient oxygen supply by difussion. North et al. (2009) used chemical biology in zebrafish to show that the blood circulation itself is required at these early stages for HSC development. Returning to the other chemicals identified in the HSC screen, several compounds were found to be modulators of heartbeat and blood flow (North *et al*., 2009). While a chemically diverse set of compounds, in general, compounds that increased blood flow through vasodilation increased HSC formation, and compounds that decreased blood flow through vasoconstriction decreased HSC formation. Emphasizing the importance of vigorous blood circulation on HSC formation, the zebrafish *silent heart *mutant that lacks a heartbeat and bloodflow, had reduced HSC formation. While chemically enhanced blood flow can increase HSC formation, only one compound, the nitric oxide (NO) donor S-nitroso-N-acetyl-penicillamine (SNAP) could increase HSC formation before the onset of blood flow. Likewise, treatment before the onset of blood flow with the NO inihibitor N-nitro-L-arginine methyl ester (L-NAME) reduced HSC formation. NO is a gaseous signaling molecule that regulates angiogenesis and vascular tone, and is a toxic antibiotic secreted by phagocytes in the immune response [68]. NO production increases in response to sheer stress and blood flow [69], and remarkably, SNAP treatment of *silent heart *mutant zebrafish embryos could rescue HSC development. Importantly, the role of NO in HSCs is conserved in mice, and loss of *Nos3 *reduces HSCs and transplantable HSCs [70]. These studies have direct relevance for clinical patients undergoing stem cell transplants: enhanced blood flow or increased NO signaling might enhance HSC production and engraftment, improving the outcome for transplant patients.

## Screening for chemical modifiers of oncogene-induced cell fate changes

Many chemotherapeutic drugs target the proliferating bulk of a cancer cells. However, even with intense chemotherapy many cancers return, suggesting that there is a less proliferative cancer cell population that can survive treatment. For the majority of patients with acute myelogenous leukemia (AML) the disease recurs within two years of treatment, and less than 10% of adults with AML patients will survive beyond five years http://www.cancerhelp.org.uk/. AML can be caused by the oncogene fusion AML1-ETO (AE), and AE redirects cells from an erythrocytic fate to a granulocytic blast cell fate [71]. Small molecules that suppress the action of this AE-induced fate change may enhance the effects of chemotherapy when combined with anti-proliferative drugs. Peterson and colleagues developed a transgenic line that expressed *AE *from the heatshock inducible promoter, and screened for small molecules that suppressed the action of AE on the erythrocytic cell lineage [72]; Figure 2E). When grown at 28.5°C, the *hsp:AML1-ETO *transgenic fish had normal blood development, and expressed *gata1 *in the posterior blood island [72]. When shifted to 40°C, *AML1-ETO *was expressed, and within an hour after heatshock, *gata1 *was no longer detectable by RNA *in situ *hybridization. After screening 2000 bioactive compounds from the Spectrum library, a COX-2 inhibitor restored *gata1 *expression without affecting the expression of the transgene. Inihibition of COX enzymes both enhanced *gata1 *expression (erythrocyte lineage), and decreased the AE driven *mpo *expression (granulocytic lineage). Notably, these effects could be reversed by the addition of dmPGE2, the major effector metabolite of COX in zebrafish. Peterson and colleagues then used morpholino oligonucleotides to directly test the genetic role of the COX enzymes. Interestingly, the AE cell fate change was strongly affected by the specific loss of the COX-2 proteins, suggesting that COX-2 is essential for the AE oncogene-induced cell fate change. This effect may be *via *transcriptional control, as AE expression in human myelogenous leukemia cells is almost five times higher than in control cells, and the cells preferentially differentiate into the myeloid lineage: an effect that can be prevented upon addition of a COX-2 inhibitor. Because PGE2 is important in stimulating β-catenin expression in the pathogenesis of cancers, such as colon cancers, the effects of AE were tested on ß-catenin expression. Importantly, AE expression dramatically enhanced β-catenin expression in human myelogenous leukemia cells, an effect that could be suppressed by chemical inhibition of COX-2. This pathway is conserved in zebrafish, as knockdown of β-catenin genes in zebrafish embryos compromised the AE differentiation activity, while treatment with a β-catenin activator, an inhibitor of the GSK-3β, enhanced the AE differentiation activity. Taken overall, this study identifies COX-2 small molecule modifiers of the AE oncogene driven cell fate changes, and identifies an AE induced COX-2-PGE2-β-catenin pathway that contributes to dysregulated hematopoietic differentiation [72]. The conservation of this pathway in fish and human cells, suggests that this pathway may be relevant to target in AML pre-clincial mammalian models.

## Using cancer drugs to restore developmental processes: Cardio-facio-cutaneous syndrome

Rare developmental disorders are unlikely to attract drug development programmes. However, if the developmental mutation is in a pathway that is mutated in common diseases, such as cancer, there may be the potential to test available drugs in the context of developmental diseases. Children with germ-line mutations in KRAS, BRAF, MEK1 and MEK2 develop Cardio-facio-cutaneous syndrome (CFC), characterized by abnormal heart, craniofacial, and skin development. Activation by the fibroblast growth factors (FGFs) leads to activation of the RAS-RAF-MEK-ERK (MAPK) pathway kinases, ultimately directing the cellular action, including proliferation, apoptosis, differentiation or senescence [73]. The MAPK pathway is one of the most frequently mutated pathways in cancer, and is the focus of intense drug development [74]. Intriguingly, the BRAF CFC syndrome mutations are both kinase-active and kinase impaired *in vitro*. Our laboratory recently expressed a panel of CFC syndrome and melanoma alleles in the developing zebrafish, and found that all alleles are gain of function mutations *in vivo*, and promote an early cell movement phenotype [75]. The highly specific and clinically active MEK inhibitor, CI-1040 was able to suppress the cell movement defect in early embryogenesis caused by CFC kinase mutations. CI-1040 is a non-ATP-competitive inhibitor, that selectively inhibits the activity of MEK1 and MEK2 in *in vitro *and *in vivo *mouse tumor models [76]. Unlike in cancer cells, a developing animal requires MAPK signaling in specific cell types, within discrete time points in development. Therefore, despite the gain of function mutations in CFC syndrome, it would not be desirable to completely inhibit MAPK signaling in the developing embryo. Indeed, treatment with CI-1040 can restore cell movements in gastrulation, but causes severe effects later in development. Treating CFC-zebrafish embryos within a specific one-hour treatment window early in zebrafish gastrulation restored normal cell movements without additional drug induced developmental defects [75]. This work highlights the intrinsic value of the zebrafish system to test drugs designed for common diseases, such as cancer, within a different disease context.

## Finding novel small molecule modulators of the FGF-MAPK pathway in zebrafish

Uncontrolled MAPK pathway activation is associated with disease and abnormal development, and the pathway is carefully attenuated by phosphatases that limit signalling. One phosphatase, the dual-specificity phosphatase (Dusp) 6 specifically dephosphorylates the extracellular signal-related kinase (ERK). Using a transgenic line that allows for visualization of *dusp6 *expression as a biosensor for FGF signalling, Tsang and colleagues screened for small molecules that enhanced FGF signalling in the developing embryo [77]. One compound, called BCI, caused an increase in *dusp6-GFP *expression that was dependent on the activity of FGF8 signalling. As described below BCI directly inhibits Dusp6, and notably, BCI was also active in human cells, supporting conservation between the human and zebrafish MAPK pathway and its regulatory enzymes. In mice, loss of *dusp6 *leads to an enhanced heart size, but the mechanism behind this heart size control is unknown [78]. In zebrafish, knockdown of *dusp6 *in development leads to early embryonic defects, which obscures the study of *dusp6 *in the heart. With a Dusp6 inhibitor in hand, the timing and cellular action of Dusp6 in heart development could be carefully studied and mapped. BCI treatment at the one to eight somite stage of development led to the expansion of myocardial progenitors, coupled with a reduction of the endothelial lineages [77]. Thus, live embryo screening of a transgenic zebrafish line has identified a specific inhibitor of the Dusp6 phosphatase and can reveal novel insight into the role of MAPK signalling during development, and possibly MAPK diseases.

## Screening for new small molecules that control zebrafish behaviour

By 2020, it is projected that major depression will become the disease responsible for the most years of disability worldwide, with bipolar disorder sixth and schizophrenia ninth (Lopez and Murray, 1998). Many behaviour-altering drugs were discovered by chance in the 1940s and 50s, and have become the prototypes for newer analogues used today [79]. There is an unmet clinical need for new classes of neuroactive molecules to treat the range of mental illnesses, and chemical tools to explore neurobiology research [79]. One of the reasons for the lack of newer neuroactive drugs comes from the lack of available relevant model systems for screening large numbers of active compounds. The complex networks of the brain cannot be modelled *in vitro*, and the expense and ethical issues surrounding mice and rats do not make them easily amenable to high throughput screening [79]. In the first high-throughput chemical screens for behaviour phenotypes, Peterson, Schier and colleagues have developed screening platforms that allow for high throughput screening of known and novel small molecules for behavioural phenotypes in living zebrafish embryos [80-82].

The rest/wake cycle is established as early as four-days post-fertilization in the developing larvae [81]. Like humans and other animals, zebrafish have wake and sleep-like states characterized by periods of activity and rest [81,83]. In the day, zebrafish display increased locomotor activity for longer periods, while night activity is characterized by short bouts of infrequent movements [81]. Using a tracking device, detailed measurements of zebrafish movement behaviour, including quantitative measurements of the frequency and duration of rest and waking activity, and the latency between states, was recorded for individual zebrafish larvae over three days each when exposed to one of over 5600 chemicals [82]. The multifactorial nature of the quantitative measurements could be organized into a profile for each treatment, called behavioural profiling. Hierarchical clustering of the profiles clustered the small molecules into two broad states, arousing and sedative. These studies in zebrafish are relevant to our understanding of human biology because arousing and sedative drugs generally showed a conserved neuropharmacology between zebrafish and mammals. Compounds that shared profiles often shared target pathways or therapeutic applications, indicating that behavioural profiling in zebrafish can identify and group bioactively similar compounds. Importantly, by virtue of the shared profiles with well-characterized drugs, the mode of action for poorly characterized compounds could be predicted. Behavioural profiling also identified new pathways that govern rest/wake behaviours, including a new role for the inflammatory signalling pathways and the Ether-a-go-go-related gene (ERG) potassium channel blockers. In this way, a systems biology approach to rest/wake neuropharmacology in zebrafish may be directly applicable to the development of new drugs for the proportion of the population (> 10%) who suffer from chronic sleep disturbances.

In another study, Peterson and colleagues have discovered a novel phenotype in zebrafish that display stereotypic motor behaviours before, during and after exposure to a high-intensity light stimulus, called the photomotor response (PMR) [80]. Using tracking devices, the PMR and an embryonic touch response (ETR) were analysed for behavioural phenotypes when exposed to 14,000 different small molecules. Behavioural information could be quantified, and organised into behavioural "barcodes". Hierarchical clustering of the barcodes revealed groups of compounds that induced similar phenotypes, called phenoclusters. Often, phenoclusters were enriched for compounds of a similar chemical class, and point to the cellular targets within a pathway. As with the chemical screening for changes in the rest/wake cycle, hierarchical clustering of the PMR and ETR phenotype could also reveal the mode-of-action for uncharacterized compounds, and provide testable hypotheses about the target identification for that compound. For example, two unrelated compounds, STR-1 and STR-2 have no known activity in mammals, and have the same behavioural barcode profile as compounds known to inhibit acetylcholinesterase (AchE). *In vitro*, STR-1 could inhibit AchE, but STR-2 required bioactivation in the embryo to inhibit AchE. Thus, phenoclustering revealed novel compounds, and accurately predicted their mechanism of action. Importantly, molecules that require activation within the context of the animal would have been missed in *in vitro *designed screens. Finally, high throughput behavioural screening may also be able to identify compounds that alter a chemical or genetic behavioural phenotype. As an example, small molecules that cause paralysis or excitation in the zebrafish embryo could be treated with chemical antidotes to restore normal behaviour. Given the unmet need for new drugs to treat mental illness and the quantity of information obtained for thousands of compounds on just a few zebrafish behaviours, high throughput behavioural screening in zebrafish has the potential to reveal important new drug leads and targetable pathways for these and more complex behaviours.

## Target identification and validation

While many currently used clinical drugs have no known target [3], target identification remains an important aspect of chemical biology. For example, to identify the target of the small molecule BCI described above, Tsang and collegues used purified Dusp6 to directly test for *in vitro *ERK phosphatase assays in the presence or absence of BCI. Computational BCI docking simulations showed an accessible crevise in Dusp6 but not Dusp5, and by binding this site, BCI acts via an allosteric mechanism to prevent the shift from low to high enzyme activity upon ERK binding [77]. In other examples, a systems approach led to the identification of the mode of action for novel neurobiological compounds [80,82]. Hierarchical clustering of the behaviour phenotypic profiles enriches for compounds that act on a similar target or target pathway, and targets of previously uncharacterized compounds have been identified by virtue of their similar behavioural phenotype with well characterized compounds. In another systems approach, we are using a combined yeast and zebrafish approach to identify the intended and unintended targets of small molecules *in vivo* (Ishizaki et al., DMM, in press).

Affinity chromatography using immobilised small molecules is another method for target identification. However, immobilisation of a small molecule through the attachment of an adequate linker can often unintentionally cause reduced activity of the compound. To address this issue, Chang and colleagues designed a 1536 triazine-tagged compound library, incorporating the linkers prior to screening to provide a straightforward method of isolation of the target compound [84]. In one screen, a triazine-tagged library was screened for enhanced pigmentation in developing zebrafish [85,86]. A compound called PPA was identified that could enhance pigmentation in both the fish embryo and also in human albino melanocytes [85,86]. Affininty chromatography identified the F1F0-ATPase as a cellular target of PPA. Notably, although PPA was identifed in the zebrafish system, it is also effective in a range of mammalian melanocyte and melanoma cells. Ion gradients appear to play a role in pigmentation, and PPA may prove a valuable research tool to study how mitochondrial ATPases control melanin in both zebrafish and mammalian melanocyte cells.

Zebrafish can play an important role in the drug development process by testing for action *in vivo*, and in structure-activity profiling. For example, Lum and colleagues screened 200,000 chemicals for Wnt/β-catenin pathway modulators using a Wnt pathway responsive reporter construct expressed in mouse L cells [64]. One class of compounds, called inhibitors of the Wnt response (IWR), specifically reduced β-catenin levels and stabilized a component of the β-catenin destruction complex, called Axin. Wnt signaling is required for zebrafish tail fin regeneration, and to test the activity of the IWR compounds *in vivo*, adult zebrafish tail fins were clipped, and treated with IWR compounds. The IWR compounds prevented tail fin regeneration as well as decreased proliferation in the gastrointestinal crypt cells, showing that Wnt signaling is critical for stem cell activities *in vivo*. Almost all colorectal cancers have activated Wnt signaling caused by mutations in the Wnt suppressors, adenomatosis polyposis coli gene or axin, or activating mutations in β-catenin, but there are currently no Wnt inhibitors in clinical trials. Novel Wnt inhibitors such as these may provide therapeutically relevant compounds, and zebrafish are playing a central role in determining their *in vivo *efficacy, structure-activity relationships, and tissue specific sensitivity [64,87].

Finally, zebrafish can provide new insight into how drugs work in an organism. Thalidomide was widely prescribed in the 1950s and 1960s in many countries, including Canada and the United Kingdom, to pregnant women suffering from morning sickness. This resulted in the birth of over ten thousand children with serious developmental birth defects, including severe shortening or absence of limbs, ear defects and other heart and gastrointestinal abnormalities. While the teratogenicity of thalidomide is well established, the mechanism behind the developmental defects is unknown. This is important because thalidomide is still used today as a treatment for multiple myeloma and as an immune suppressant for treating the painful leprosy asssociated erythema nodosum leprosum. Handa and colleagues identified cereblon (CRBN) and DNA binding protein 1 (DDB1) as binding partners of thalidomide in cancer cell extracts [88]. Using biochemical techniques, Handa and colleagues showed that CRBN forms a functional E3 ubiquitin ligase complex with Cullin (Cul) 4 and DDB1; importantly, thalidomide binding to CRBN inhibits E3 function. Ultimately, thalidomide may have multiple targets in a developing organism, but chemical and genetic approaches in zebrafish showed CRBN to be a relevant *in vivo *target of thalidomide in limb outgrowth. Unlike mice and rats, that are insensitive to thalidomide teratogenicity, zebrafish embryos treated with thalidomide show otolith and angiogenic deficiencies and fail to develop outgrowth of pectoral fins [88,89]. Gene knockdown of *crbn *or *cul4 *in zebrafish caused a loss of the developing fin, and fin development could be rescued by a thalidomide-insensitive mutant form of *crbn*. Together, this evidence points to the binding and inhibition of Crbn as the casue of the teratogenic effect of thalidomide in the ears and limbs. The E3 targets of Crbn are unknown, but expression of Fgf8 at the apical ectodermal ridge of the zebrafish fin bud was dramatically reduced upon thalidomide treatment, a phenotype that could also be rescued by the thalidomide-insensitive mutant form of *crbn. *The thalidomide-Crbn-Fgf8 pathway is conserved in the chick limbs, providing evidence that the zebrafish limb phenotypes are relevant in other species. Identification of the dangerous teratogen targets can aid in generation of thalidomide deriviatives that no longer inhibit Crbn E3 activity, and the sensitivity of zebrafish to thalidomide will be a valuable living tool for screening new thalidomide deriviatives.

## Conclusions

The combination of genetic and developmental features places zebrafish small molecule screening at the cutting edge of chemical biology. Ten years after the first small molecule screen in zebrafish, we have examples of how small molecules can lead to fundamental insight into developmental and behavioural processes, to new clinical strategies, and to understanding of the action of currently used drugs. In addition to the chemical biology examples presented here, other important screens in zebrafish have identified the first regulators of the BMP pathway [90,91], regulators of TGFβ signaling [92], histone deacetylase inhibitors that can suppress models of polycystic kidney disease [93] and cancer cell radiosensitizers [94], among others. We are only just beginning to understand how small molecules act within living animals and the transparent nature of the zebrafish embryo may facilitate future visualization and understanding of how chemicals act upon targets within cells. As high throughput screening becomes more accessible, a greater range of chemical space within biological systems can be explored. Finally, sharing of chemical libraries between zebrafish researchers with diverse biological interests should lead to an unprecedented wealth of new insight into chemical biology within a whole animal system [95].

## Competing interests

The authors declare that they have no competing interests.

## Authors' contributions

This review article was written by KLT, NDT and EEP, and all artwork was designed and illustrated by NJG. All authors read and approved the final manuscript.
